# Maize Gene Atlas Developed by RNA Sequencing and Comparative Evaluation of Transcriptomes Based on RNA Sequencing and Microarrays

**DOI:** 10.1371/journal.pone.0061005

**Published:** 2013-04-23

**Authors:** Rajandeep S. Sekhon, Roman Briskine, Candice N. Hirsch, Chad L. Myers, Nathan M. Springer, C. Robin Buell, Natalia de Leon, Shawn M. Kaeppler

**Affiliations:** 1 Department of Agronomy, University of Wisconsin, Madison, Wisconsin, United States of America; 2 Department of Energy Great Lakes Bioenergy Research Center, University of Wisconsin, Madison, Wisconsin, United States of America; 3 Department of Computer Science and Engineering, University of Minnesota, Minneapolis, Minnesota, United States of America; 4 Department of Plant Biology, Michigan State University, East Lansing, Michigan, United States of America; 5 Department of Energy Great Lakes Bioenergy Research Center, Michigan State University, East Lansing, Michigan, United States of America; 6 Microbial and Plant Genomics Institute, Department of Plant Biology, University of Minnesota, Saint Paul, Minnesota, United States of America; Wuhan University, China

## Abstract

Transcriptome analysis is a valuable tool for identification and characterization of genes and pathways underlying plant growth and development. We previously published a microarray-based maize gene atlas from the analysis of 60 unique spatially and temporally separated tissues from 11 maize organs [1]. To enhance the coverage and resolution of the maize gene atlas, we have analyzed 18 selected tissues representing five organs using RNA sequencing (RNA-Seq). For a direct comparison of the two methodologies, the same RNA samples originally used for our microarray-based atlas were evaluated using RNA-Seq. Both technologies produced similar transcriptome profiles as evident from high Pearson's correlation statistics ranging from 0.70 to 0.83, and from nearly identical clustering of the tissues. RNA-Seq provided enhanced coverage of the transcriptome, with 82.1% of the filtered maize genes detected as expressed in at least one tissue by RNA-Seq compared to only 56.5% detected by microarrays. Further, from the set of 465 maize genes that have been historically well characterized by mutant analysis, 427 show significant expression in at least one tissue by RNA-Seq compared to 390 by microarray analysis. RNA-Seq provided higher resolution for identifying tissue-specific expression as well as for distinguishing the expression profiles of closely related paralogs as compared to microarray-derived profiles. Co-expression analysis derived from the microarray and RNA-Seq data revealed that broadly similar networks result from both platforms, and that co-expression estimates are stable even when constructed from mixed data including both RNA-Seq and microarray expression data. The RNA-Seq information provides a useful complement to the microarray-based maize gene atlas and helps to further understand the dynamics of transcription during maize development.

## Introduction

Knowledge of the genome, transcriptome, proteome, and metabolome is the basis of systems biology, a holistic approach that strives to understand the role and interaction of individual components in shaping the phenotype. Revolutionary genomic technologies developed over the past decade have resulted in the generation of rich information about the genomes and transcriptomes of many species. This has led to increased efforts to understand gene function and expression networks that vary temporally and spatially through development of an organism. In plants, transcriptome profiles during development have been documented in several species including Arabidopsis (*Arabidopsis thaliana*) [2], maize (*Zea mays*) [1], rice (*Oryza sativa*) [3], soybean (*Glycine max*) [4], barley (*Hordeum vulgare*) [5], and Medicago (*Medicago truncatula*) [6].

Traditionally, genome-wide transcriptional analysis has been performed using microarray technologies. For instance, some of the early work in maize was based on spotted cDNA amplicons [7]–[10], a technology which was replaced by spotted oligonucleotide arrays [11]. With the development of *in situ* DNA synthesis approaches, platforms such as Affymetrix served as the primary tool for transcriptomic analysis [12]–[14]. Transcriptome analysis efforts have been further enabled by the completion of reference genomes [15]. Previously, we utilized the maize genome sequence to design a custom NimbleGen array and develop a gene atlas that documents expression patterns through maize development [1].

While microarrays have been very useful for transcriptome analyses, there are some inherent drawbacks of this technology. First, due to the static nature of microarray-based expression data, expression can only be determined for gene models included on the array. For genome sequences such as maize that are evolving in terms of both gene content and gene model structural annotation, the use of microarrays could result in missing expression information for a substantial number for genes not available or annotated at the time of array design. Second, reliance of microarray technology on DNA-DNA hybridization can potentially lead to inaccurate expression estimates for genes sharing high sequence homology. Finally, due to background noise and signal saturation, the dynamic range of expression obtained from microarrays is limited and, therefore, may not be suitable for comparing very highly or lowly expressed genes.

The development of high-throughput “next-generation” sequencing technologies has enabled the use of RNA sequencing (RNA-Seq) as an attractive alternative to microarrays for transcriptome analyses [16]. A major advantage of RNA-Seq technology is flexibility since the data can be reanalyzed to obtain updated information as the genome sequence and annotation evolves, and the stringency of mapping parameters can be adjusted to potentially discern expression of highly homologous genes. In addition, with the cost of sequencing decreasing in recent years, this technology is rapidly decreasing in cost. Several recent studies have exploited this technology to generate transcriptome information for many plant species including Arabidopsis [17], rice [18], [19], and soybean [4], [20]. In maize, RNA-Seq has been used to develop detailed transcriptome information for leaf [21] and inflorescence [22], [23].

In this study, we used RNA-Seq to obtain expression profiles for samples previously profiled using a microarray [1] to enhance the resolution in expression of gene family members and to permit assessment of expression across the entire genome. We performed several analyses to compare the effectiveness of the two technologies in providing genome-wide gene expression estimates. We also examined the quality of co-expression networks developed from the two data sets. This data is available to the community to serve as a complementary resource to the microarray-based expression atlas.

## Materials and Methods

### Plant materials, growing conditions and RNA extraction

Remnant total RNA from a subset of samples used for the microarray-based maize gene atlas [1] was used for this experiment. The tissue samples were obtained from reference inbred line B73 plants grown at the West Madison Agricultural Research Station (Verona, WI) during summer 2008. Growing conditions, sampling method, and detailed description of the samples and other relevant information are as previously described [1]. The list of samples included in this study is provided in Table 1.

**Table 1:** List of tissues included in RNA-Seq-based gene atlas.

#	Tissue name	Plant ontology term	Plant ontology tissue description
1	24H_Germinating Seed	PO:0009001	Fruit (Kernel)
2	6DAS_GH_Primary Root	PO:0020127	Primary root
3	V3_Stem and SAM	PO:0020148	Soot apical meristem
		PO:0020142	Stem internode
4	V5_Tip of stage-2 Leaf	PO:0025142	Leaf tip
		PO:0009025	Vascular leaf
5	V9_Immature Leaves	PO:0009025	Vascular leaf
6	16DAP_Endosperm	PO:0009089	Endosperm
7	16DAP_Embryo	PO:0009009	Plant embryo
8	V9_Eighth Leaf	PO:0009025	Vascular leaf
9	V9_Eleventh Leaf	PO:0009025	Vascular leaf
10	V9_Thirteenth Leaf	PO:0009025	Vascular leaf
11	VT_Thirteenth Leaf	PO:0009025	Vascular leaf
12	R2_Thirteenth Leaf	PO:0009025	Vascular leaf
13	10DAP_Whole seed	PO:0009001	Fruit
14	12DAP_Whole seed	PO:0009001	Fruit
15	12DAP_Endopsperm	PO:0009089	Endosperm
16	14DAP_Whole seed	PO:0009001	Fruit
17	14DAP_Endopsperm	PO:0009089	Endosperm
18	16DAP_Whole seed	PO:0009001	Fruit

H, hours; DAS, days after sowing; GH, greenhouse; V, vegetative; DAP, days after pollination; VT, vegetative tasseling; R, reproductive.

### Calculation of RNA-Seq expression values

From approximately 5 µg of total RNA, mRNA was isolated, fragmented, converted to cDNA, and PCR amplified according to the Illumina RNA-Seq protocol (Illumina, Inc. San Diego, CA). Sequence reads were generated using the Illumina Genome Analyzer II (San Diego, CA) and Illumina HiSeq 2000 (San Diego, CA) at the University of Wisconsin Biotechnology Center (Madison, WI). Illumina barcodes were used to multiplex a portion of the samples. Sequence reads generated were between 35 and 101 bp single-end reads. Sequencing platform, multiplexing, and read length for each sample can be found in Table S1. RNA-Seq read quality was evaluated based on the Illumina purity filter and distribution of base quality scores at each cycle. All data presented passed the quality control filtering based on these metrics. Sequences are available in the Sequence Read Archive at the National Center for Biotechnology Information (accession number SRP010680).

Sequence reads for each tissue were mapped to v1 and v2 of the B73 reference pseudomolecules (http://ftp.maizesequence.org/) [15] using Bowtie version 0.12.7 [24] and the splice site aware aligner TopHat version 1.2.0 [25]. The minimum and maximum intron length was set to 5 bp and 60,000 bp respectively; all other parameters were set to the default values. Gene model annotation was not provided during the read mapping. Normalized gene expression values expressed as fragments per kilobase pair of exon model per million fragments mapped (FPKM) were determined using Cufflinks version 0.9.3 [26]. The maximum intron length was set to 60,000 bp and the quartile normalization option was used. For the alignments to the v1 pseudomolecules, the 4a.53 annotation (http://ftp.maizesequence.org/) was provided as the reference annotation and the v1 pseudomolecules were provided for the bias detection and correction algorithms. For the alignments to the v2 pseudomolecules, the 5b annotation (http://ftp.maizesequence.org/) and v2 pseudomolecules were provided. The default settings were used for all other parameters. An average of FPKM value of three replicates was used for all the analyses.

### Microarray and RNA-Seq correlations

Microarray and RNA-Seq expression values were based on mapping the probes (microarray) or reads (RNA-Seq) to version 4a.53 for direct comparison of these analyses with the microarray data set published earlier [1]. In case of multiple annotated transcripts per genes, a transcript encoding the longest peptide was chosen. Since the microarray design did not cover all the 4a.53 gene models due to lack of a pseudomolecule assembly at the time of design, this comparison was based on 22,151 genes common between the microarray and RNA-Seq data sets. Correlations were calculated for all 22,151 genes as well as for a subset of genes that were expressed in both data sets. For the correlation estimates based only on expressed genes, 19,744 genes with average expression value of at least 200 in one of the 60 tissues included in earlier study [1] were selected from the microarray dataset. For RNA-Seq data set, genes with an FPKM 95% confidence interval lower boundary greater than zero [27], as defined by Cufflinks [26], in at least one of the tissues were designated as transcribed and chosen for correlation analysis. Based on this criterion, 1,933 genes from the set of 19,744 genes expressed in microarray were not expressed in RNA-Seq data set and hence removed. In all, 17,811 unique transcripts were used for this analysis. Log_2_ transformed values were used for correlation calculations. To avoid taking the log of a number less than 1, all such FPKM values were replaced by 1.

### Principal Component Analysis

Principal Component Analysis (PCA) was performed using the Spotfire DecisionSite for Functional Genomics (DSFG) package (http://spotfire.tibco.com/). FPKM values and RMA-normalized log_2_-transformed expression values were used for the RNA-Seq and microarray data, respectively. To avoid taking the log of a number less than 1, all such FPKM values were replaced by 1. The analysis involved k-means clustering in order to group genes into 1000 clusters followed by PCA.

### Hierarchical clustering

Hierarchical clustering was performed using the unweighted pair-group method with complete linkage approach and Pearson's correlation as a similarity measure in the Spotfire DSFG package (http://spotfire.tibco.com/).

### Coexpression network analysis

Genes that did not have detectable expression levels in either dataset were removed leaving 19,328 filtered gene set (FGS) genes for further analysis. For the microarray dataset, genes with the average expression value exceeding 200 in at least one of the tissues were considered expressed. In case of RNA-Seq, the average expression value of a gene had to be greater than 0 FPKM in at least one of the 18 tissues. Due to the differences in dynamic ranges of the two platforms, we applied log_2_ transformation to the microarray expression data and inverse hyperbolic sine transformation to the RNA-Seq data. The latter compresses larger values more than smaller values and works well for the values below 1. Individual coexpression networks were generated based on the transformed datasets by calculating Pearson correlation coefficient for each pair of gene expression profiles using Sleipnir library [28]. Fisher transformation and normalization were applied to the values in both coexpression networks [29]. Expression conservation scores were derived by calculating Pearson correlation coefficient for each pair of gene coexpression profiles [30]. The significance of EC score was determined based on the gene's null expectation derived from the bootstrapping analysis that involved generation of 1,000 random co-expression network pairs by selecting a mixture of RNA-Seq and microarray profiles for the 18 tissue samples.

## Results and Discussion

### Overview of samples and quality assessment

We used RNA-Seq to profile the transcriptome of 18 tissues representing distinct stages of maize plant development. These tissues are a subset of samples included in a microarray-based gene atlas described previously [1]. For direct comparison of the two technologies, remnant total RNA from the microarray study was used for RNA-Seq. A complete list, brief description, and plant ontology terms of the samples included in this study are provided in Table 1. For each sample, total RNA from three biological replicates, each composed of pooled tissue from three randomly chosen plants, was subjected to sequencing. For each tissue, we generated between 5 and 28 million single-end (35–101 bp) reads averaged across all three replicates (Table S1). Of these, 55.8 to 88.8% of the reads were mapped to the B73 filtered gene set transcripts (version 5b; www.maizesequence.org) and expression values in units of fragments per kilobase of exon model per million fragments mapped (FPKM) was calculated. While multiple transcripts have been predicted for the majority of maize genes, for this analysis, we selected the transcript encoding the longest peptide to represent each gene. The biological replicates were highly correlated (Figure S1), with an average Pearson's correlation coefficient between replicates of 0.971±0.004 with 83% of the correlations over 0.950 (Table S2). These observations affirmed the technical reproducibility of the RNA-Seq technology and reproducibility of biological replicates despite having variable read numbers, read lengths, and percentage of reads mapped across the tissues and biological replicates.

### Global gene expression trends

For all analyses performed on a gene basis, we worked with the transcripts from the FGS that exclude transposons, pseudogenes, contaminants, and other low-confidence annotations. Genes with an FPKM 95% confidence interval lower boundary greater than zero [27], as defined by Cufflinks [26], were designated as transcribed in the RNA-Seq data set. Based on this criterion, 29,447 (74.7%) of the 39,429 genes were transcribed in at least one tissue. Of the non-expressed genes, 18.3% are *ab initio* genes predicted by Fgenesh [31] which accounted for 60.1% of all *ab initio* genes in the current version (5b) of the maize genome. Similarly, 22.3% of the non-expressed genes encode for transcripts with size below 500 bp, which account for 67.6% of all such genes. In contrast, only 0.2% encode for transcripts larger than 5 kb. Finally, 84.4% of the non-expressed genes lack functional annotation according the current data (www.maizesequence.org); these account for 42.6% of all non-annotated genes in the genome. Thus, while some of these genes likely represent those not expressed in the tissues included in this study, others might be poorly annotated. However, some of these genes might encode rare transcripts that were missed due to lower sequencing depth. This is suggested by higher number of non-expressed genes observed in V9_Immature Leaves and 16DAP_Endosperm (Figure S2); both these tissues have lowest number of reads (Table S1).

Classification of transcribed genes on their magnitude of expression showed substantial variation in the range of expression among tissues (Figure S2). Variation in expression was also evident from distribution of FPKM values for all genes (Figure S3A) and expressed genes (Figure S3B). To further investigate the representation of genes among tissues, a subset of highly expressed genes for each tissue were identified (Table S3) which, in many cases, tended to have specific biological activities characteristic of that tissue. For instance, consistent with initiation of a period of high increase in fresh weight of endosperm at 16 Days After Pollination (DAP), eight out of ten highly expressed genes at this stage encoded zein proteins. Zeins are the major seed storage proteins that account for roughly half of the endosperm proteins [32] and their up-regulation is consistent with dramatic endsperm growth and accumulation of storage compounds at this stage [33]. However, none of the zein-encoding genes were represented among the ten highest expressed genes in 12DAP endosperm in which a gene encoding defensin was the highest expressed gene. Defensins are antimicrobial proteins that protect the seed and developing embryo against pathogens infections [34]. Interestingly, consistent with higher oil accumulation in maize embryos, a gene encoding oleosin - structural proteins found in vascular plant oil bodies - was most abundant in 16DAP embryo. Likewise, genes encoding proteins involved in photosynthesis were over-represented in mature leaves e.g. eighth leaf at vegetative 9 (V9) stage and thirteenth leaf at vegetative tasseling (VT) stage. For such tissues with predominance of a specific biological activity, a large proportion of reads might represent the abundantly expressed genes and, therefore, deeper sequencing will be needed for detection of genes with relatively low expression levels.

**Figure 1:**
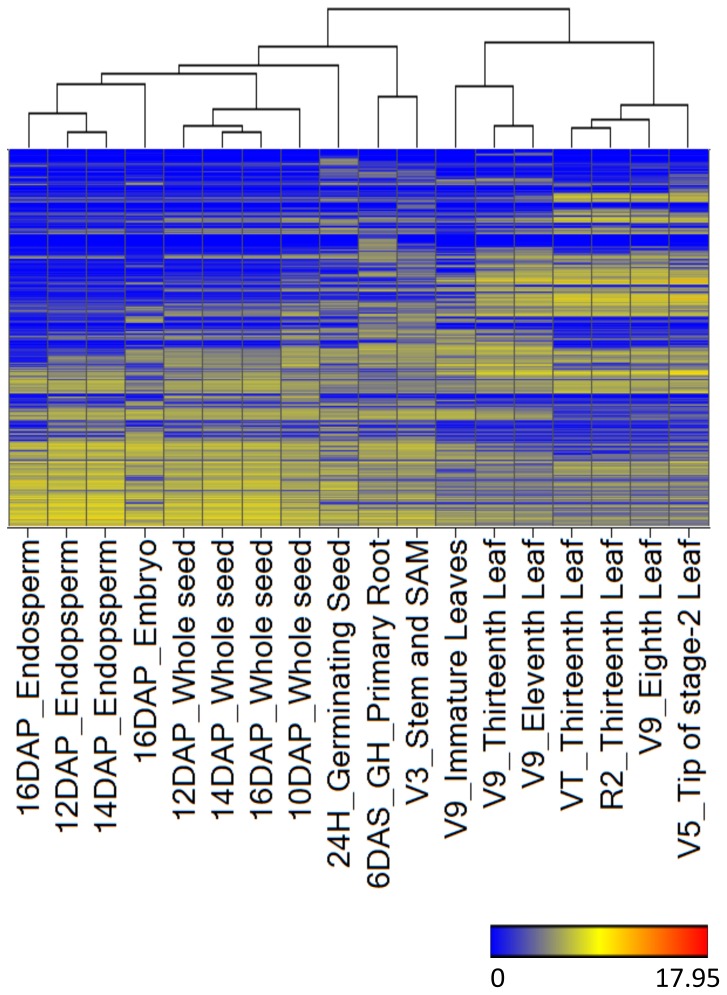
Heat map showing hierarchical clustering of tissues based on global gene expression. Clustering was based on log_2_-transformed Fragments Per Kilobase Exon model per Million mapped fragments (FPKM) values of 29,038 genes that were detected in at least one tissue based on the FPKM 95% confidence interval lower boundary greater than zero. Red, yellow, and blue colors indicate high, medium, and low levels of gene log_2_-transformed expression, respectively.

Biological identity of the tissues was well reflected in the RNA-Seq-based transcriptome analysis as revealed by hierarchical clustering (Figure 1). A distinct cluster of 6 Days After Sowing (DAS) primary root containing root apical meristem and V3 stem and shoot apical meristem (SAM) indicate commonalities in the transcriptome of meristematic tissues. Germinating seed and embryo each had a distinct transcriptome consistent with the specialized biological function of these tissues. Transcriptional differences among organs at different developmental stages were also well captured as exemplified by leaf tissues. Maize leaf, a developmentally complex organ, has been divided into at least three stages of active cell division and growth (designated as stages I, II, and III) followed by a fully mature state, with each of the stages having distinct morphological and anatomical features [35]. For instance, the transcriptome of V9 immmature leaves, which belong to stage II characterized by rapid blade and ligule growth [35], was quite distinct from V9 eighth leaf, a fully mature leaf (Figure 1).

While the depth of sequencing for some tissues was somewhat low (Table S1), very high correlations between biological replicates together with clustering of the tissues based on their biology indicates that the sampling depth is sufficient for drawing inferences about the transcriptome.

### RNA-Seq and microarrays produce very similar global expression trends

**Figure 2:**
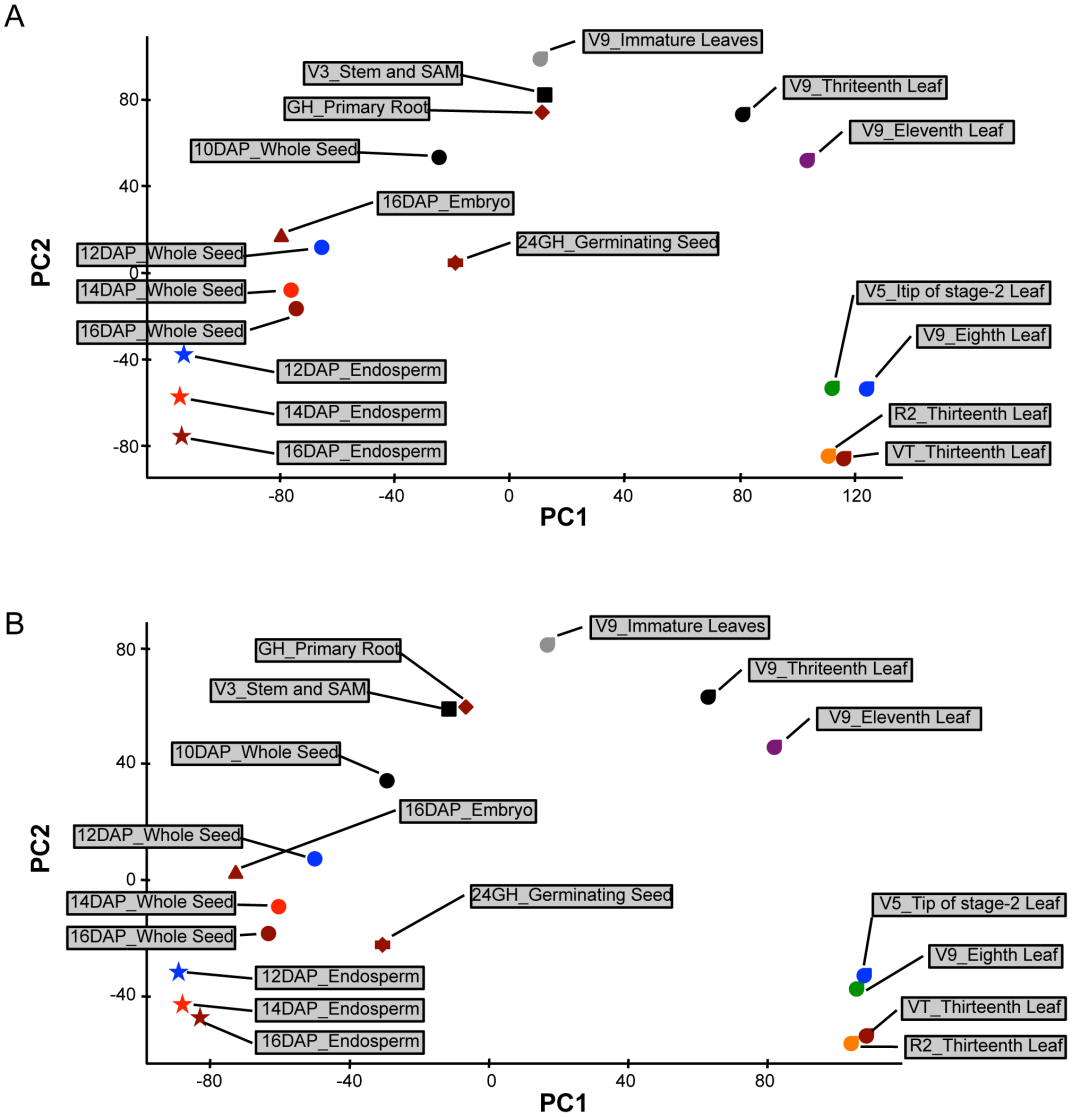
Principal Component Analysis (PCA) showing similarities between transcriptome profiles produced by RNA-Seq (A) and microarray (B). PCA was performed independently for both datasets. First principal component (PC1) is shown on x-axis while the second principal component (PC2) is shown on y-axis. Tissues belonging to same organ group are represented by different colors of the same shape.

We compared the RNA-Seq expression dataset with the previously published microarray based gene atlas [1] for various aspects of transcriptional analyses. The fact that both data sets were generated from the exact same RNA samples eliminated the variance due to growing conditions, tissue handling, and RNA extraction. For all comparisons, we used the microarray and RNA-Seq expression data generated based on the 4a.53 annotation for consistency as this was the version used for the microarray-based gene atlas [1]. Since microarray design covered only 22,151 genes, only these genes were used for calculating Pearson's correlation coefficients between tissues. Correlation estimates were computed for all 22,151 genes as well as for only a subset of genes that were expressed in both data sets (See Materials and Methods). Gene expression estimates for the eighteen tissues from RNA-Seq and microarray were significantly (P<0.001) correlated with the Pearson's correlation coefficients ranging between 0.70 and 0.83 (Table 2, Figure S4). The correlation estimates based on all common genes as well as those based only on the expressed genes were very similar (Table 2). These correlation estimates are similar to those reported earlier [23], [36], [37]. For further comparison, we evaluated the effectiveness of both techniques in categorizing tissues based on global gene expression using PCA performed independently for RNA-Seq and microarray datasets. Interestingly, both technologies produced very similar tissue clusters that reflected biological relatedness and developmental stage of the tissues (Figure 2). For instance, seed and leaf tissues were separated by the first principal component owing to their distinct developmental profiles by both RNA-Seq and microarray (Figure 2 A and B). Similarly, whole seed and endosperm tissues were also separated by the second principal component in both analyses, wherein the differences can be attributed to transcriptomes of the embryo and pericarp – two tissues that differentiate these samples. To summarize, these analyses showed that RNA-Seq and microarray transcriptome profiles are highly correlated.

**Table 2:** Correlations between RNA-Seq and microarray-based expression values.

#	Tissue	*r* (Pearson) (For genes expressed in both platforms)	*r* (Pearson) (For all common genes for both platforms)
1	24H_Germinating Seed	0.73	0.71
2	6DAS_GH_Primary Root	0.71	0.72
3	V3_Stem and SAM	0.67	0.71
4	V5_Tip of stage-2 Leaf	0.77	0.78
5	V9_Immature Leaves	0.75	0.77
6	V9_Thirteenth Leaf	0.71	0.74
7	V9_Eleventh Leaf	0.69	0.72
8	V9_Eighth Leaf	0.72	0.75
9	VT_Thirteenth Leaf	0.75	0.76
10	R2_Thirteenth Leaf	0.75	0.77
11	10DAP_Whole seed	0.75	0.76
12	12DAP_Whole seed	0.79	0.79
13	14DAP_Whole seed	0.80	0.78
14	16DAP_Whole seed	0.80	0.81
15	12DAP_Endosperm	0.83	0.81
16	14DAP_Endosperm	0.83	0.82
17	16DAP_Endosperm	0.81	0.81
18	16DAP_Embryo	0.79	0.79

### RNA-Seq based gene atlas provides better breadth of coverage of the transcriptome compared to the microarray-derived atlas

To compare the effectiveness of RNA-Seq and microarray technologies in transcriptome analyses, we compared the RNA-Seq data set with microarray-based gene atlas [1]. Of the 32,535 gene models (4a.53), 82.1% were detected in at least one tissue by RNA-Seq following the criterion described above and previously used [27], while only 56.5% were detected by microarray using a cutoff expression value of 200 as described previously [1]. Primarily, this disparity is due to lower coverage of the microarray platform as only 22,153 (68.1%) of the gene models (4a.53, see Materials and Methods) were represented on our custom NimbleGen microarray. Nevertheless, RNA-Seq clearly provided a more comprehensive picture of the transcriptome. RNA-Seq also provided better sampling of the classical maize genes that have been historically identified based on striking mutant phenotype and overrepresented in maize genetics literature [38]; of 464 classical genes, 427 were detected by RNA-Seq compared to 390 by the microarray. Furthermore, expression patterns of these genes followed expected trends. For instance, *brown midrib3* (*bm3*), which encodes caffeic acid O-methyltransferase enzyme involved in the lignin biosynthetic pathway [39], was predominantly expressed in developing leaves concomitant with active lignification (Figure S5). Expression of glossy15, an APETALA2-like gene that controls juvenile to adult vegetative phase change [40], was expressed only in shoot apical meristem at vegetative-3 stage. Interestingly, expression of DMT101, the closest homolog of the Arabidopsis MET1 gene [41], showed a developmental gradient in endosperm that was highest at 14DAP. This is consistent with notion that endosperm is the most likely target tissue for genomic imprinting and that imprinting is associated with DNA hypermethylation [42]. Finally, expression of *purple plant1*, which encodes a *Myb* transcription factor that controls anthocyanin synthesis in leaves and sheaths [43], was expressed specifically in these tissues.

**Figure 3:**
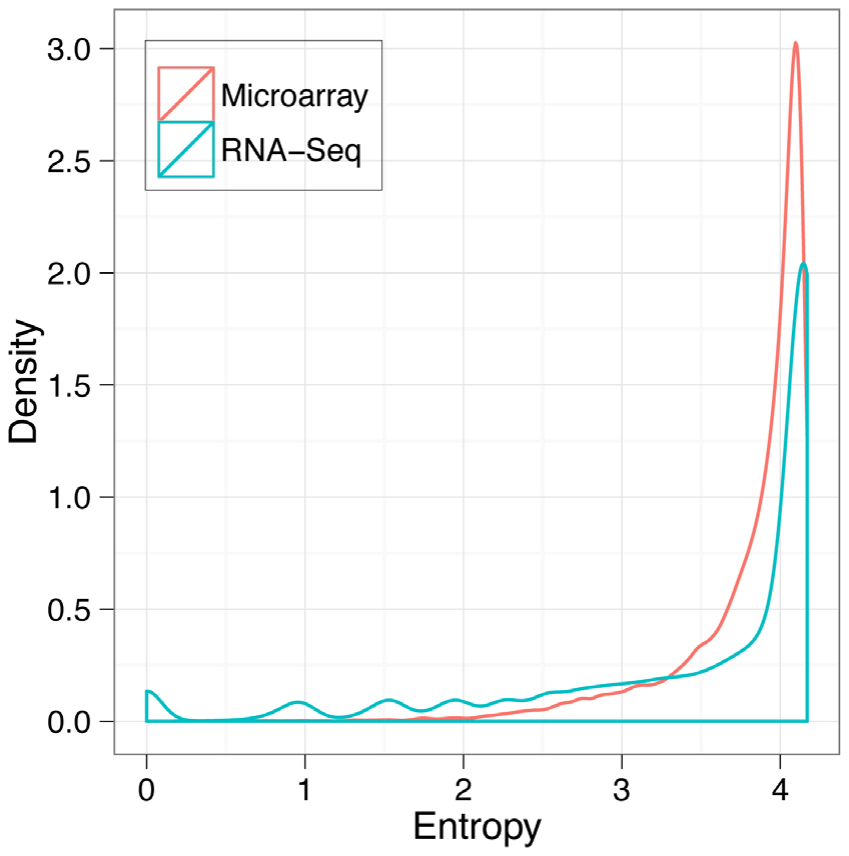
Shannon entropy was calculated for each gene expression profile to assess their tissue specificity. Distribution of the entropy values is shown for both Microarray and RNA-Seq datasets. Tissue-specific expression patterns are more prevalent in the RNA-Seq dataset (Mann-Whitney U test, p<0.01) indicating higher sensitivity of the platform to the expression differences between genes.

Shannon entropy [44], [45] is often used to estimate the tissue-specificity of gene expression across samples. The tissue-specificity of gene expression was assessed in both platforms, and there were more examples of tissue-specific patterns in RNA-Seq data than in microarray data (Mann-Whitney U test, p<0.01; Figure 3). Thus, RNA-Seq provided enhanced coverage of the transcriptome with more tissue-specific patterns.

### Resolution of expression of paralogs by RNA-Seq and microarray

We compared the two technologies for discerning expression of paralogs. There were a total of 2,434 paralog pairs for which we had both microarray and RNA-Seq expression data. These pairs include genes from the two sub-genomes of maize resulting from a whole-genome duplication event [46]. The correlation of the expression levels for the two genes was assessed within each platform and was compared to random pairs of genes. Analysis of RNA-Seq expression data showed that paralogs were highly correlated compared to random pairs of genes (Figure S6). However, there were more examples of highly correlated pairs of paralogs (>2 standard deviations above random gene pairs) within the microarray data (41.2%) than within the RNA-Seq data (31.1%). This higher correlation between paralogs within the microarray data is expected as the microarray was not always specifically assessing expression of individual genes.

**Figure 4:**
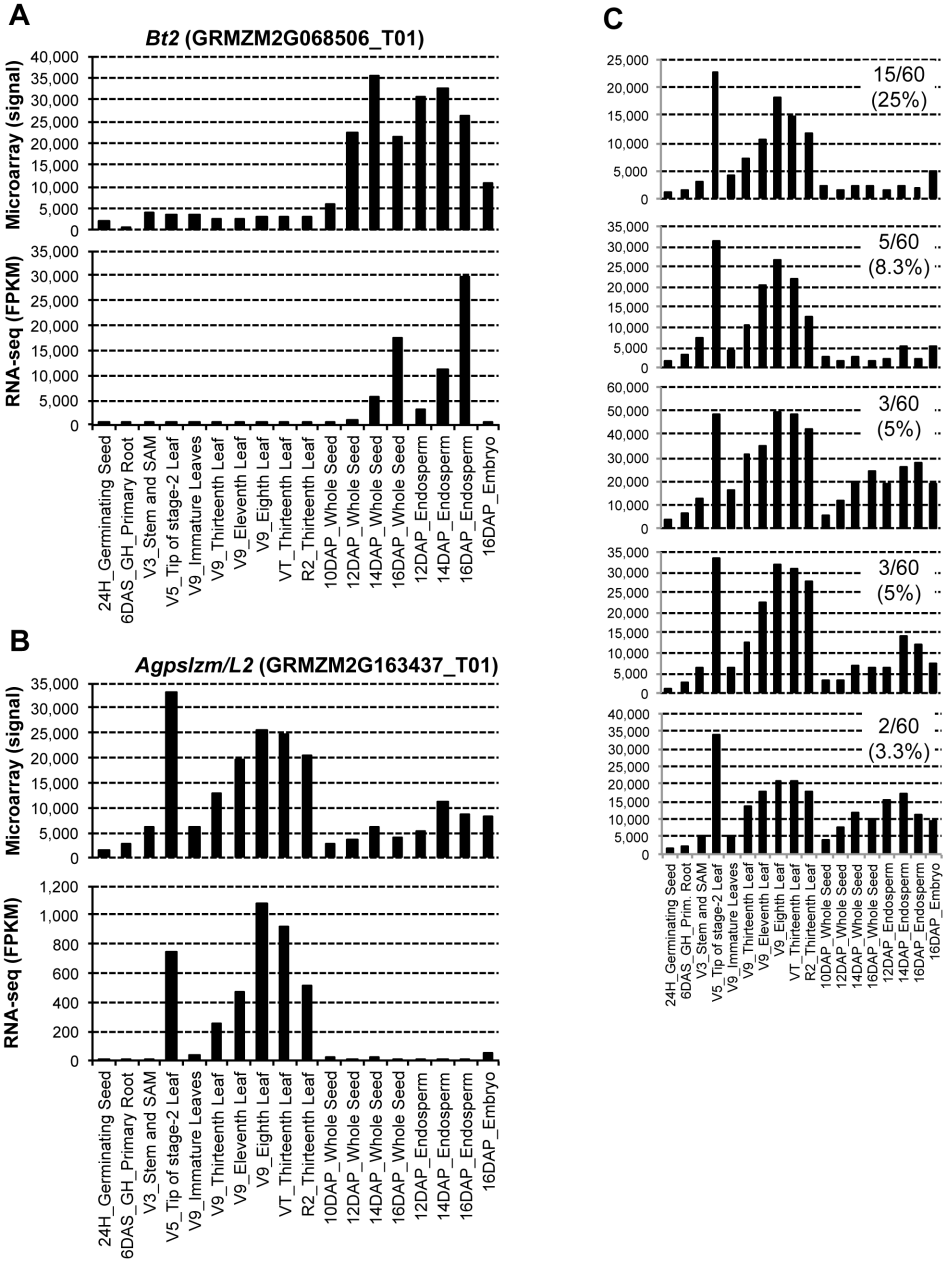
Relative efficiencies of RNA-Seq and microarray in discerning expression of two paralogous genes. A. Expression patterns of endosperm-specific *Brittle-2* (*Bt2*) gene B. Expression patterns of leaf specific *Agpslzm/L* gene. C. Expression patterns of five individual probes representing *Agpslzm/L* gene. Sequence differences of each of the 60-mer probes from the paralogous *Bt2*, shown as number of mismatches, are in the inset of each graph.

As a specific example, we chose two paralogous genes, *Brittle-2* (*Bt2*) [47] and *Agpslzm*/*L2* [48] which both encode a small subunit of ADP-glucose pyrophosphorylase (AGP). The two genes share high nucleotide similarity at the mRNA level (84%) and likely arose during tetraploidization of maize genome [49]. However, the two genes are tissue-specific; *Bt2* encodes a cytosolic small subunit and is expressed in the endosperm while *Agpslzm*/*L2* encodes a plastidial small subunit and is expressed in leaves [49], [50]. Based on microarrays, the expression of endosperm-specific *Bt2* was substantially higher in endosperm and whole seeds, but detectable levels of expression were also observed in some of the leaf samples (Figure 4A). Furthermore, expression was also detected in embryo which could actually be contributed by *Agp2*, the third gene encoding a plastidial AGP small subunit specific to embryo [51]. Using RNA-Seq, however, expression was strictly limited to the seed tissues. Likewise, microarrays detected substantial expression of the leaf specific *Agpslzm*/*L2* in seed tissues while with RNA-Seq, detectable expression was only found in mature leaves which are expected to accumulate starch (Figure 4B). To determine if spurious expression in microarray data is due to cross-hybridization of the probes, we examined the expression of individual probes representing *Agpslzm*/*L2* gene. Indeed, the *Agpslzm*/*L2* probes with 2–3 mismatches out of 60 nucleotides with *Bt2* produced sizable spurious signal in seed tissues, and only with 5 or more mismatches did the *Agpslzm*/*L2* expression became specific to seed (Figure 4C). Therefore, it appears that cross hybridization is an important contributing factor for lower resolution of paralog expression in microarrays. Based on this data, RNA-Seq clearly provides better resolution of expression of genes with similar sequence.

### Similarities and differences in RNA-Seq and microarray co-expression networks

The RNA-Seq and microarray transcriptome profiles from 18 samples were used to generate co-expression networks to assess how the profiling platform affected network properties. A set of 19,328 FGS genes that demonstrated detectable expression in the microarray profiles and had mapped reads in at least one RNA-Seq sample were used for this analysis. The two expression profiling platforms have different dynamic ranges, which can complicate comparisons of the data. The microarray data was log_2_ transformed while the RNA-Seq data was log transformed using an inverse hyperbolic sine function, which allows for greater compression of the larger values that are present in RNA-Seq data. Co-expression networks were generated for the averaged microarray and RNA-Seq data for the 18 tissues.

**Figure 5:**
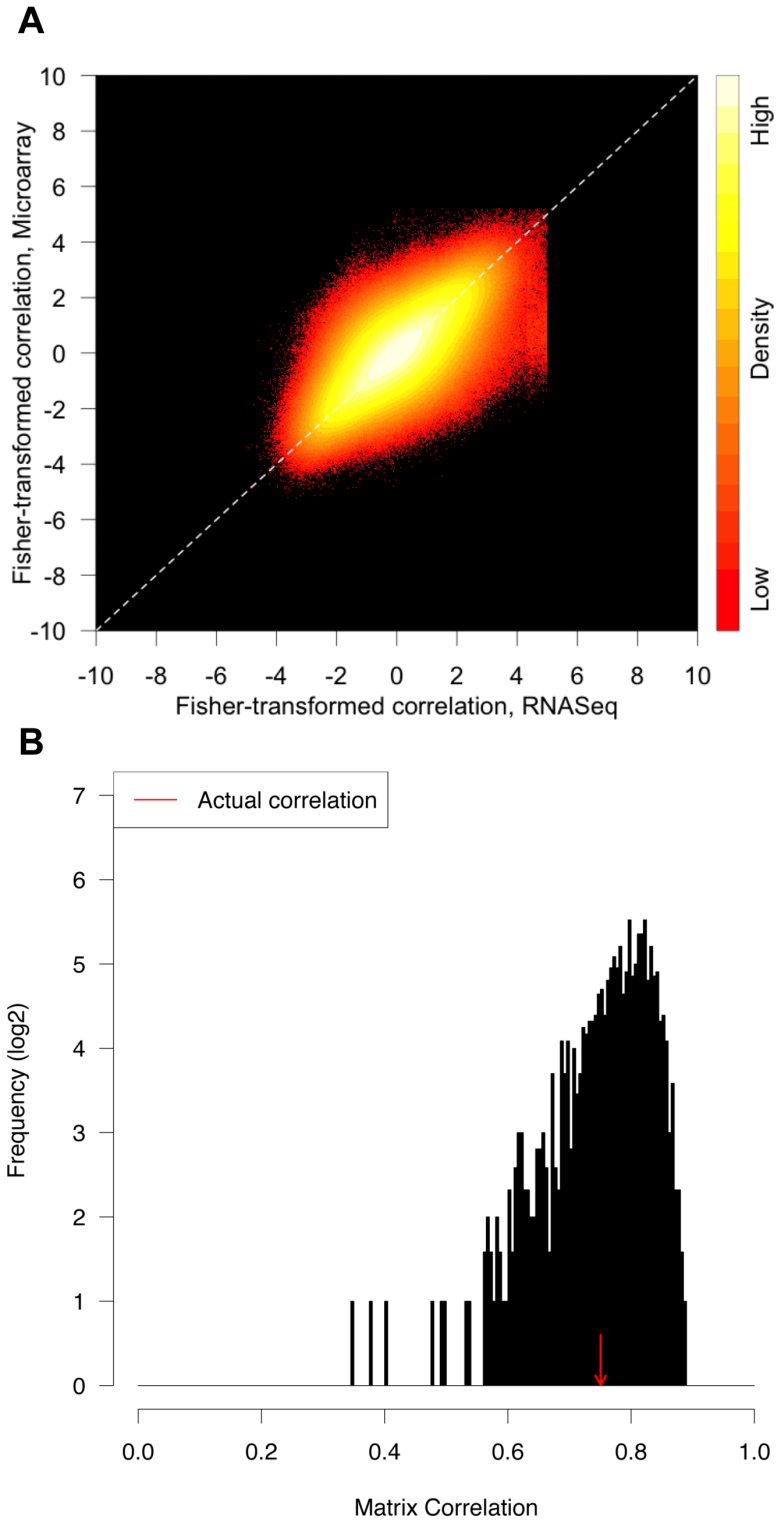
Comparison of RNA-Seq and microarray co-expression networks. (A) The density of Fisher-transformed and normalized edge weights are shown for both the microarray (y-axis) and RNA-Seq (x-axis) co-expression networks. (B) The frequency of correlation coefficient (R) values for a series of 1000 random co-expression networks is plotted relative to the observed value (red arrow). The random co-expression networks were generated by selecting a mixture of RNA-Seq and microarray data for each of the two networks.

In general, the two networks contain many examples of similar co-expression relationships and exhibit a relatively high global correlation of R = 0.75 (Figure 5A). However, this correlation between the networks was slightly lower than the correlation between co-expression networks generated from two biological replicate samples of microarray data (R = 0.86) or RNA-Seq data (R = 0.90). The reduced similarity between the RNA-Seq and microarray networks was partially driven by a large set of gene-pairs that exhibit near perfect correlation (R = 1) in the RNA-Seq network but a range of correlations in the microarray network (indicated by the cluster of points below and to the right of the dotted line in Figure 5A). We investigated a set of 1000 genes connecting a large fraction of these edges and found that they exhibited significantly lower mean RNA-Seq counts than the rest of the genome (Mann-Whitney U test, p<1e-55). Many of these co-expression relationships may be false-positives due to spurious correlations among genes with very low expression levels. Indeed, a more stringent criterion requiring FPKM >5 in at least one tissue removed 841 genes from the analysis and resulted in slightly improved matrix correlations for the microarray and expression data (R = 0.78) (not shown). This observation suggests that caution should be used when computing correlation for genes with very low coverage in RNA-Seq. Microarrays do not typically provide significant co-expression relationships for these genes, likely because the background noise accompanying the low intensity signal prevents measurement of spurious correlations.

We further explored the impact of computing co-expression networks from datasets composed of a mix of RNA-Seq and microarray expression profiles as compared to data from a single platform. A series of 1,000 pairs of co-expression networks were generated by randomly forming two groups of profiles, each composed of a mixture of RNA-Seq and microarray profiles for the 18 samples (Figure 5B). The similarity between each pair of co-expression networks generated from mixed data was compared to the observed similarity between the networks from pure RNA-Seq and microarray datasets. The observed similarity between the single-platform networks falls within the range of values for the mixed networks (Figure 5B), suggesting that robust co-expression networks could be generated from a mixture of RNA-Seq and microarray profiles.

**Figure 6:**
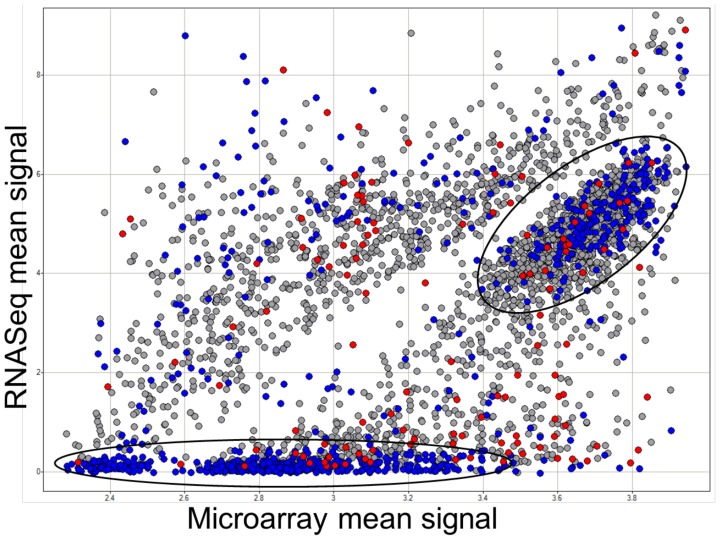
Comparison of expression profiles for individual genes in RNA-Seq and microarray co-expression networks based on expression conservation. The 3,354 genes with significant differences (p<0.01) in expression conservation between RNA-Seq and microarray data were assessed. The mean expression level in microarray samples (x-axis; log_2_ tranformed) and RNA-Seq samples (y-axis; inverse hyperbolic sine transformed) was compared. The color coding indicates connectivity in the two co-expression networks; red indicates the 122 genes with more connections in the microarray network, blue indicates the 796 genes with more connections in the RNA-Seq co-expression network and grey indicates relatively similar connectivity in both networks. The circles indicate two clusters of genes with divergent EC scores.

Expression conservation (EC) provides one method for assessing the similarity of co-expression relationships for individual genes in two different networks. An EC score is based on a comparison of a gene's neighbors in two different networks. Genes with significantly different EC scores show different patterns of co-expression, i.e. different neighbors, in the two networks. An analysis of EC scores computed for the RNA-Seq and microarray-derived co-expression networks revealed that the majority of genes (82.6%) have similar neighbors in both networks. However, there are 3,354 genes with significant differences, based on the EC score measure (p<0.01). We investigated several features of these genes to understand the factors that might contribute to divergent co-expression relationships between the two platforms. The genes with divergent EC values are enriched for genes with retained duplicates in the two sub-genomes (p<0.05). There was no evidence for significant enrichment for genes in one of the two sub-genomes [52]. A comparison of the mean expression levels for genes with significantly different co-expression relationships revealed several major groups of genes with distinct expression level characteristics (Figure 6). One group of divergent genes is highly expressed in both platforms. However, within the microarray data, these genes are clustered around the maximum measurable expression value in some tissues and are likely expressed outside the dynamic range of microarrays. Therefore, one possibility is that the co-expression relationships derived from RNA-Seq data capture more information for these genes given the increased dynamic range on that platform. The other group of genes with divergent EC scores has very low expression levels in RNA-Seq data and a range of expression values in microarray data. In fact, most of these genes have a median expression level of zero in the RNA-Seq samples indicating that over half of the samples lack expression of the gene. The majority of these genes have significant EC scores due to having higher connectivity (several highly correlated partners) in the RNA-Seq data. Using a stringent criterion requiring FPKM>5 in at least one tissue (see above) removed a number of genes with significant differences (not shown). As discussed previously, this suggests that caution should be used in computing co-expression for genes with low coverage in RNA-Seq.

### Availability of the maize transcriptome

To facilitate gene discovery and functional genomics of maize and related grasses, the RNA-Seq-based transcriptome is available to the community at Maize Genetics and Genomics Database (www.maizeGDB.org) MaizeGDB [53], and additional data to that described here will be incorporated as it becomes available. The dataset is linked to the B73 genome browser based on GBrowse2 (www.gmode.org) where viewers can quickly browse the expression (FPKM) of a gene of interest in all the tissues (Figure S7). Expression information is available at both the individual transcript level and the gene model level along with physical location, gene sequence, and a table of expression values. The microarray-based transcriptome [1] is also available in the same display for obtaining comprehensive expression information based on both technologies. A table of FPKM values for the tissues described in this manuscript is also provided (Table S4).

## Conclusions

In this study, we performed transcriptional analysis of 18 representative maize tissues capturing important aspects of maize development using RNA-Seq. While we reported on a microarray-based maize gene atlas [1] earlier, this data set provides enhanced coverage of the transcriptome, and provides an opportunity for comparison of RNA-Seq and microarray technologies for transcriptional analysis. A coarse comparison showed that both technologies produced a very similar overview of the transcriptome. However, RNA-Seq provides enhanced coverage of the genome as microarrays are limited by the gene models represented on the microarray chip. RNA-Seq also provided better resolution of expression differences among paralogs. Co-expression networks are highly valuable tools for identification of novel genes in biological pathways and assigning functional annotation to genes of no known function. We found that co-expression networks developed from RNA-Seq and microarrays are highly comparable. The differences in co-expression networks from the two platforms can largely be attributed to differences in the dynamic range or in the precision of estimating exact levels of expression for low-expressed genes.

## Supporting Information

Figure S1 **Quality of the biological replicates.** Pair-wise Pearson's correlation (*r*) was calculated for all three pairs of biological replicates for each tissue.
(TIF)

Figure S2 **Distribution of genes based on magnitude of expression in 18 maize tissues.** For each tissue, a gene was considered expressed if the FPKM value and FPKM lower 95% confidence interval was more than 0. For each tissue, the expressed genes were further divided in to low (FPKM >0 to ≤5), medium (FPKM >5 to ≤200), and high (FPKM >200) expression.
(TIF)

Figure S3 **Distribution of FPKM values for all genes (A) and expressed genes (B).** Expression values of all 39,429 genes and 29,447 genes were used to make the distribution plots, respectively. Since the major differences in the smaller set is absence of genes with no expression, the two plots look very similar.
(TIF)

Figure S4 **Correlations between gene expression estimates for each of the eighteen tissues obtained by RNA-Seq and microarray.** In each panel, the average (log_2_) FPKM value for each gene is shown on *x*-axis while average (log_2_) relative expression based on microarray is shown on *y*-axis. See Materials and Methods section for details of correlation analysis.
(TIF)

Figure S5 **Expression profiles of selected classical maize genes derived using RNA-Seq.**
(TIF)

Figure S6 **Density estimates for the distribution of the correlation coefficients of paralogous genes in the RNA-seq co-expression network.** The correlation co-efficients among tissues were calculated for 2,434 pairs of paralogs (from Schnable et al., 2011, PNAS) that were expressed in multiple tissues. The density plot illustrates the values for these correlation coefficients relative to a set of randomly selected genes.
(TIF)

Figure S7 **Sreenshot of the RNA-Seq data display at Maize Genetics and Genomics Database (** www.maizeGDB.org **).** Data display is based on mapping the RNA-Seq reads to version 2 of the B73 reference genome (http://ftp.maizesequence.org). Data display and download of FPKM values based on transcript and gene level is available. FPKM values were calculated using Cufflinks version 0.9.3 [26] and the 5b annotation (http://ftp.maizesequence.org).
(TIF)

Table S1 **Average number of reads, read length, and other details of sequencing.**
(XLSX)

Table S2 **Pearson's correlation coefficients for biological replicates.** All correlations had P<0.0001.
(XLSX)

Table S3 **Top 10 expressed genes in each tissue.**
(XLSX)

Table S4 **FPKM values for all transcript for all the tissues included in the study based on mapping to v2 of the B73 reference pseudomolecules.**
(TXT.ZIP)
