# Correction: Maize Gene Atlas Developed by RNA Sequencing and Comparative Evaluation of Transcriptomes Based on RNA Sequencing and Microarrays

**DOI:** 10.1371/annotation/0444d495-9231-4097-abe0-4750b9045971

**Published:** 2014-01-03

**Authors:** Rajandeep S. Sekhon, Roman Briskine, Candice N. Hirsch, Chad L. Myers, Nathan M. Springer, C. Robin Buell, Natalia de Leon, Shawn M. Kaeppler

Table S4 does not correctly download from the article. Please view the correct file for Table S4 here: 
