# Supplementary figures and images for: Maize Gene Atlas Developed by RNA Sequencing and Comparative Evaluation of Transcriptomes Based on RNA Sequencing and Microarrays
Source: PLoS One. 2013 Apr 23;8(4):e61005. doi: 10.1371/journal.pone.0061005 (PMC3634062; doi:10.1371/journal.pone.0061005)

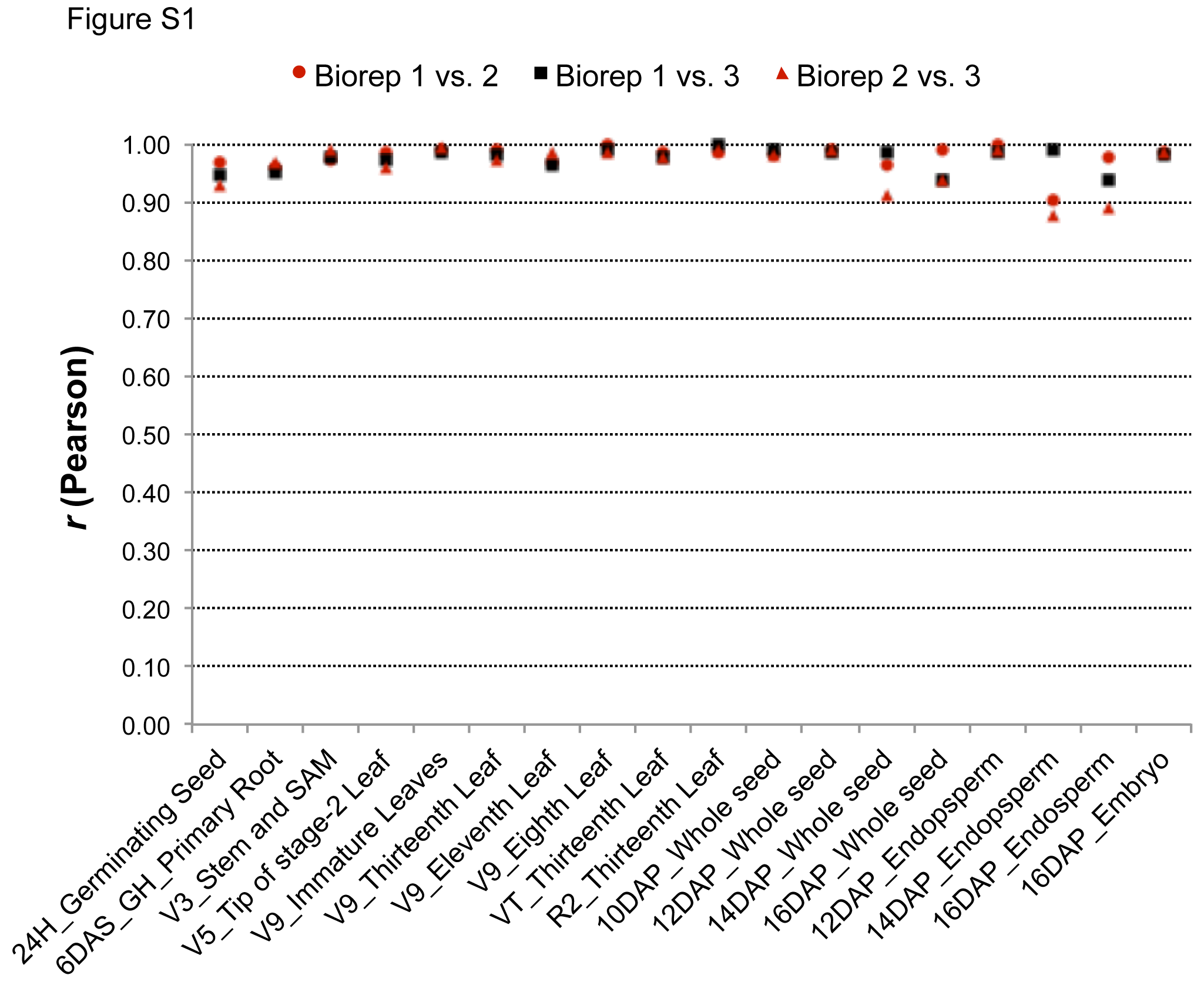

Supplement: Figure S1 — Quality of the biological replicates. Pair-wise Pearson's correlation (r) was calculated for all three pairs of biological replicates for each tissue. (TIF) [file pone.0061005.s001.tif]

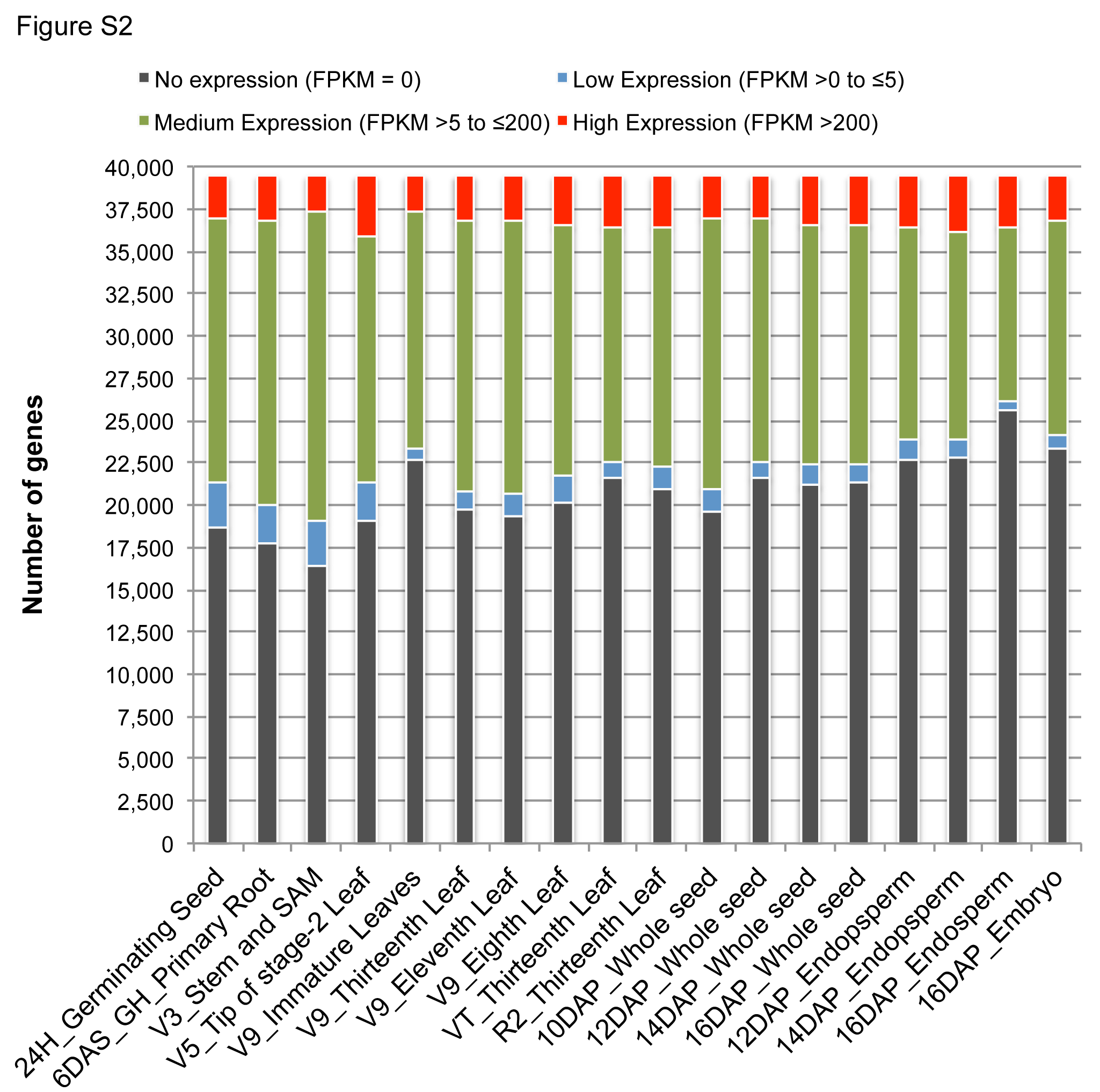

Supplement: Figure S2 — Distribution of genes based on magnitude of expression in 18 maize tissues. For each tissue, a gene was considered expressed if the FPKM value and FPKM lower 95% confidence interval was more than 0. For each tissue, the expressed genes were further divided in to low (FPKM >0 to ≤5), medium (FPKM >5 to ≤200), and high (FPKM >200) expression. (TIF) [file pone.0061005.s002.tif]

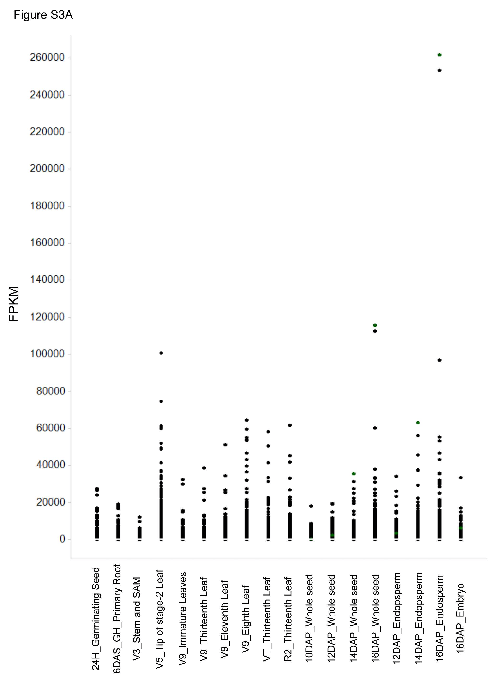

Supplement: Figure S3 — Distribution of FPKM values for all genes (A) and expressed genes (B). Expression values of all 39,429 genes and 29,447 genes were used to make the distribution plots, respectively. Since the major differences in the smaller set is absence of genes with no expression, the two plots look very similar. (TIF) [file pone.0061005.s003.tif]

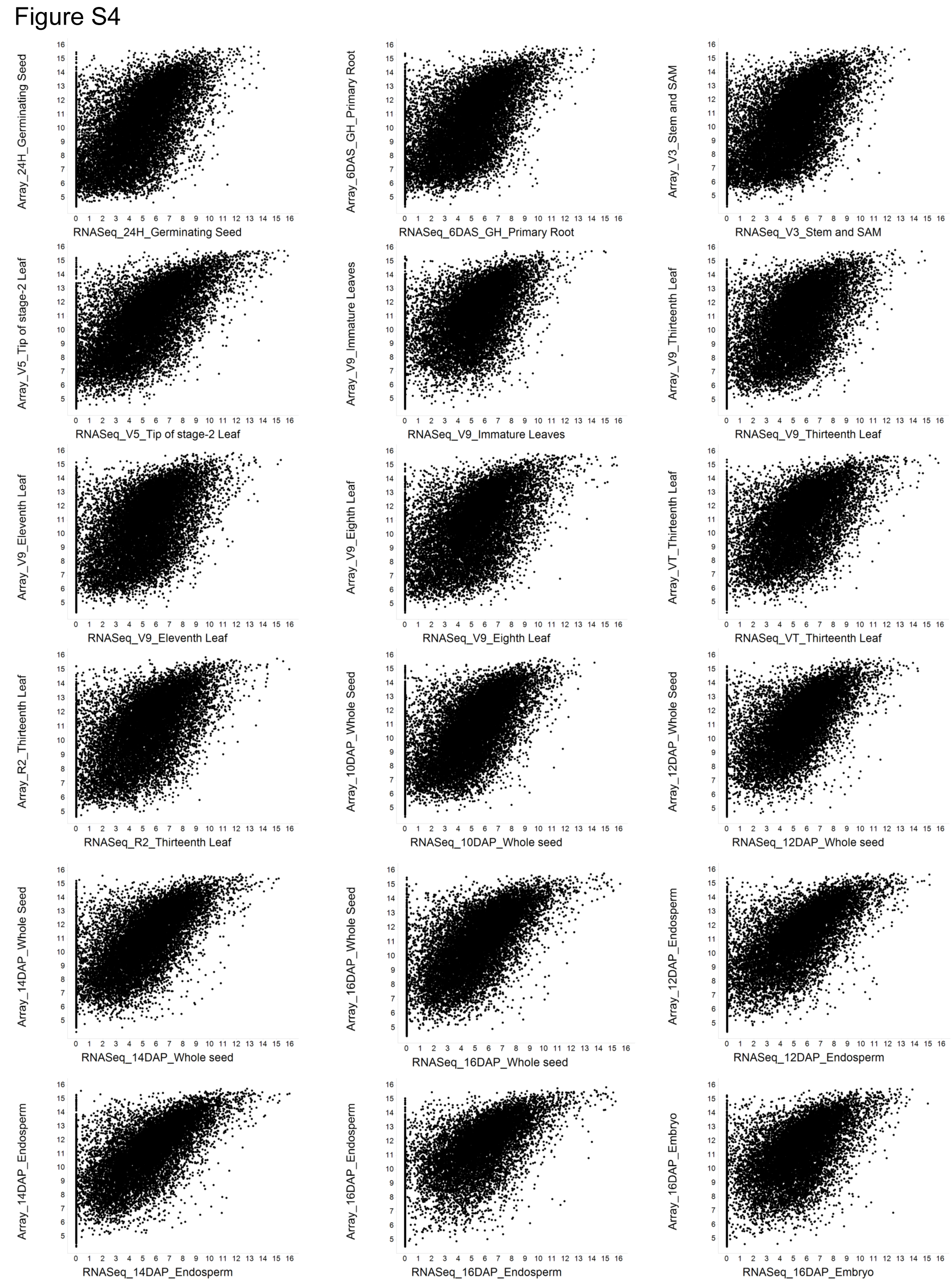

Supplement: Figure S4 — Correlations between gene expression estimates for each of the eighteen tissues obtained by RNA-Seq and microarray. In each panel, the average (log2) FPKM value for each gene is shown on x-axis while average (log2) relative expression based on microarray is shown on y-axis. See Materials and Methods section for details of correlation analysis. (TIF) [file pone.0061005.s004.tif]

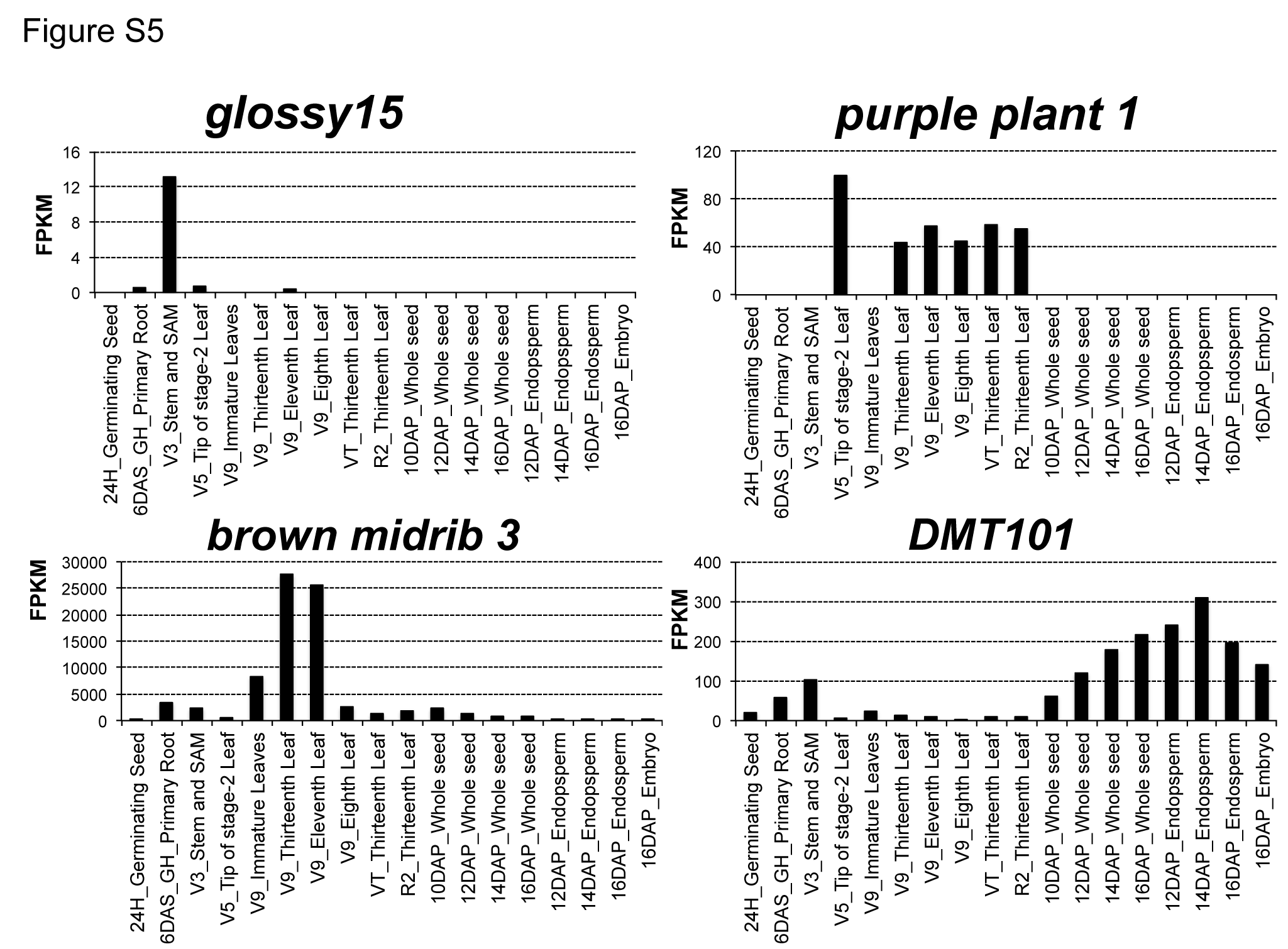

Supplement: Figure S5 — Expression profiles of selected classical maize genes derived using RNA-Seq. (TIF) [file pone.0061005.s005.tif]

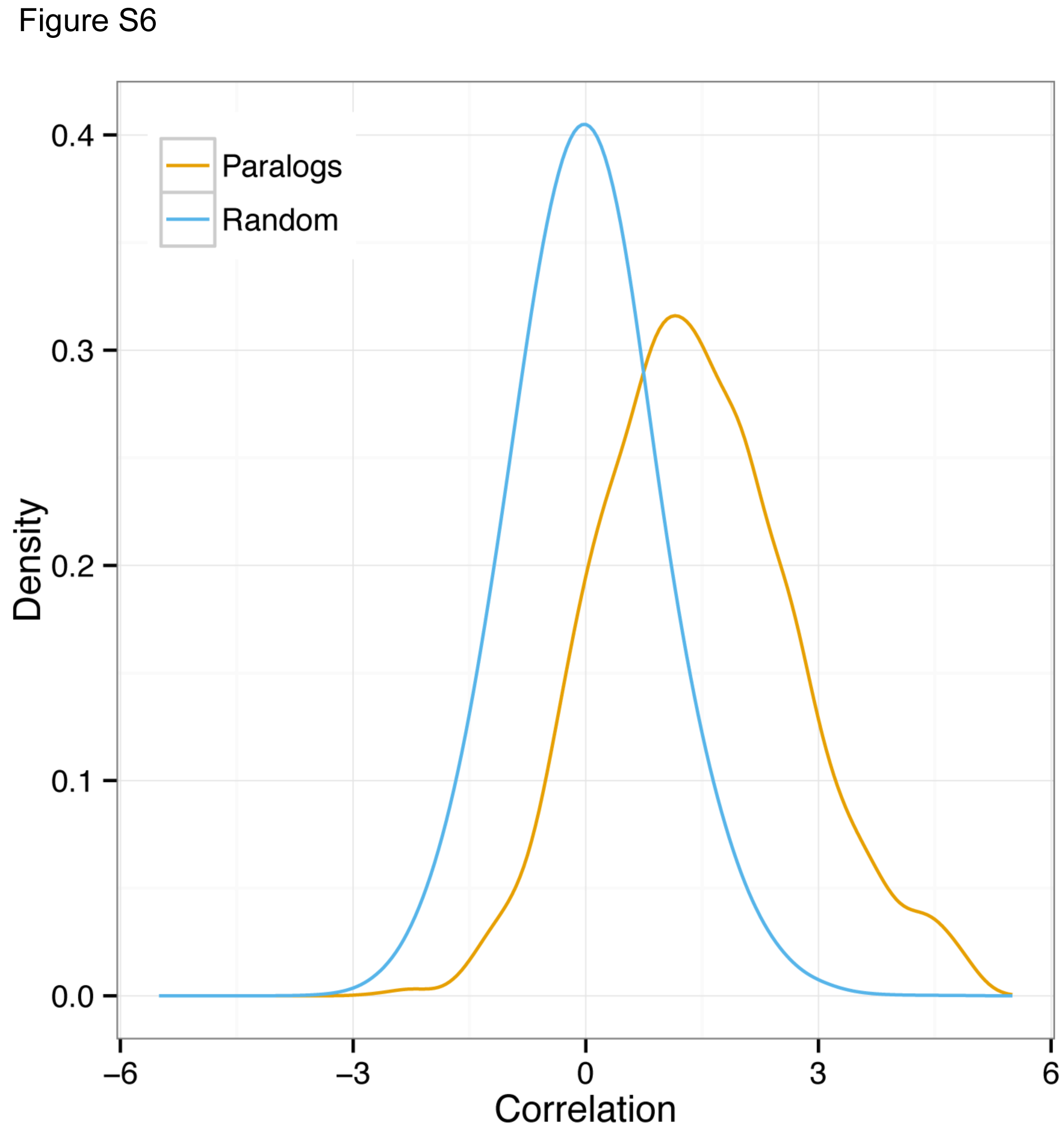

Supplement: Figure S6 — Density estimates for the distribution of the correlation coefficients of paralogous genes in the RNA-seq co-expression network. The correlation co-efficients among tissues were calculated for 2,434 pairs of paralogs (from Schnable et al., 2011, PNAS) that were expressed in multiple tissues. The density plot illustrates the values for these correlation coefficients relative to a set of randomly selected genes. (TIF) [file pone.0061005.s006.tif]

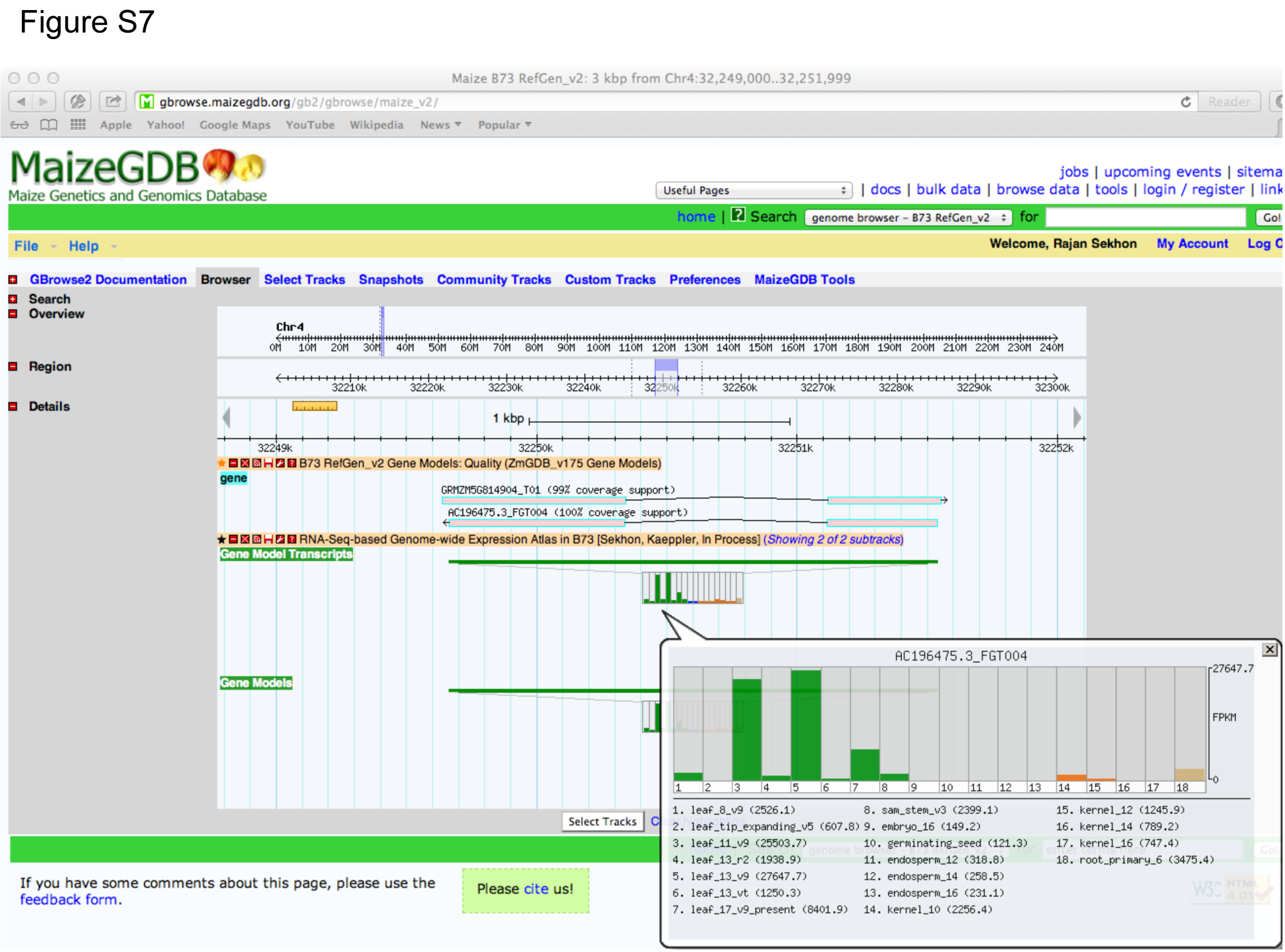

Supplement: Figure S7 — Sreenshot of the RNA-Seq data display at Maize Genetics and Genomics Database ( www.maizeGDB.org ). Data display is based on mapping the RNA-Seq reads to version 2 of the B73 reference genome (http://ftp.maizesequence.org). Data display and download of FPKM values based on transcript and gene level is available. FPKM values were calculated using Cufflinks version 0.9.3 [26] and the 5b annotation (http://ftp.maizesequence.org). (TIF) [file pone.0061005.s007.tif]
